# Differentiation of Medicinal Plants According to Solvents, Processing, Origin, and Season by Means of Multivariate Analysis of Spectroscopic and Liquid Chromatography Data

**DOI:** 10.3390/molecules28104075

**Published:** 2023-05-13

**Authors:** Lenka Burdejova, Blanka Tobolkova, Martin Polovka, Jarmila Neugebauerova

**Affiliations:** 1 Institute of Analytical Chemistry, Czech Academy of Sciences, Veveri 967/97, 602 00 Brno, Czech Republic; burdejova@iach.cz; 2 Department of Chemistry and Food Analysis, National Agricultural and Food Centre—Food Research Institute, Priemyselna 4, 824 75 Bratislava, Slovakia; 3 Department of Vegetable Growing and Floriculture, Faculty of Horticulture, Mendel University in Brno, Valticka 337, 691 44 Lednice, Czech Republic; jarmila.neugebauerova@mendelu.cz

**Keywords:** medicinal plants, solvent effect, processing and seasonal factor, geographical origin, multi-experimental analysis, multivariate statistical analysis

## Abstract

Effects of processing and extraction solvents on antioxidant properties and other characteristics were evaluated for ten medicinal plant species originating from two different localities and two production years. A combination of spectroscopic and liquid chromatography techniques possessed data for multivariate statistics. Water, 50% (*v*/*v*) ethanol, and dimethyl sulfoxide (DMSO) were compared to select the most suitable solvent for the isolation of functional components from the frozen/dried medicinal plants. DMSO and 50% (*v*/*v*) ethanol were evaluated as more efficient for phenolic compounds and colorants extraction, while water was more useful for element extraction. Drying and extraction of herbs with 50% (*v*/*v*) ethanol was the most appropriate treatment to ensure a high yield of most compounds. The satisfactory differentiation of herbs (61.8–100%) confirmed the significant effect of the processing, geographical, and seasonal factors on target functional component concentrations. Total phenolic and total flavonoid compounds content, total antioxidant activity expressed as TAA, yellowness, chroma, and browning index were identified as the most important markers for medicinal plant differentiation.

## 1. Introduction

Traditionally, medicinal plants represent an important natural source of antioxidants, particularly phenolic compounds, vitamins, carotenoids, and some metals such as Cu, Fe, Mn, and Zn [1]. Medicinal plants and their components are frequently used to improve the properties of food as well as for the development of functional foods.

Solvent extraction represents a frequently used method for the isolation of plant antioxidants. The selection of a solvent with a polarity closest to the polarity of the desired antioxidants is a crucial step to ensure that most of the compounds are extracted from the plant [2,3,4]. The commonly used solvents are ethanol, methanol, acetone, ethyl acetate, acetonitrile, and their aqueous mixtures [3,4,5,6]; occasionally used solvents are dimethyl sulfoxide (DMSO) and dimethylformamide [7,8]. Generally, binary solvents are found to be superior to the mono-solvent systems in the extraction efficiency of herbal antioxidants [9]. Water and ethanol–water mixtures are the most frequently used solvents for application in foods due to their acceptability for human consumption [10,11].

The impact of post-harvest processing on phytochemical concentration is frequently investigated. The antioxidants can be extracted from fresh, frozen, or dried plant material. Drying is the most widely used preservation method, based on moisture elimination from fresh material; thus, reducing microbial and enzymatic activity and consequently preserving the product for extended shelf life [12]. The increasing temperature during the drying has a considerable effect on the stability of some sensitive phytochemicals, which might be degraded or bio-transformed [13]. It should be noted that the drying process is considered a time/energy-consuming and expensive part of herbal production. On the other hand, it decreases the weight of medicinal plants, thus decreasing the packaging, storage, and transportation costs [14].

Freezing (i.e., storage at −18 °C) represents another preservation technique suitable for compounds of interest preservation in medicinal plants. Some authors pointed to the lowered content of phenolic compounds after the freezing, suggesting that freezing can cause damage to plant cells by the action of enzymes and substrates [1,15]. Additionally, numerous factors directly affect the quality of medicinal plants comprising genotypic factors, climate, growing conditions, agronomic, harvest, and post-harvest processing [16,17].

Due to a wider scale of methods that are usually involved in the studies aimed at medicinal plant characterization and classification from various aspects, the obtained experimental characteristics typically represent a complex matrix of variables. Thus the multivariate statistical analysis (chemometrics) is effectively utilized for sample classification, mainly in terms of variety, geographical origin, or season [1,5]. The utilization of these methods is popular in both analytical and applied chemistry. Multivariate statistics use mathematical and statistical methods to extract information from large data sets with chemical or biological information [18]. These methods allow the reduction of multidimensional and correlated data to only a few dimensions [1,5,18]. The main advantage of chemometrics is that it considers more than one factor in data analysis, i.e., it looks at the various independent variables that influence the dependent variable. The conclusions drawn from chemometric analysis are more likely to be accurate. Although there will always be errors, by considering all the possible variables, there is less chance of missing something and making an incorrect assumption [18].

In this study, a complex analysis of the influence of processing factors such as extraction solvents, post-harvest processing, as well as geographical and seasonal factors on selected qualitative parameters of medicinal plants grown in two different regions of South Moravia and harvested in two different years, was performed. Altogether, 32 herbal characteristics were evaluated and processed by the multivariate statistics to verify the possibility of herbal sample differentiation according to the above-mentioned factors, as well as to identify the most important markers for herbal differentiation. Spectroscopic and liquid chromatography techniques, in combination with multivariate statistics, were originally applied for herbal extracts differentiation and classification.

## 2. Results and Discussion

Our previous study proved that chemometric analysis based on spectroscopic and chromatographic analyses could be effectively used for the differentiation of ethanolic extracts of medicinal plants according to post-harvest treatment, plant families, and species [1]. The presented work complements this study with new data focused on other factors, e.g., effects of solvents, and geographical and seasonal aspects, affecting the herbal phytochemical and mineral composition. A complex dataset of 32 individual experimental characteristics of 228 herbal extracts (of 3 extraction solvents) has been obtained and processed. Detailed information on individual experimental characteristics of medicinal plant extracts as affected by solvent type, post-harvest processing, locality, and season are summarized in Supplementary Table S1.

### 2.1. Effect of Extraction Solvents on Characteristics of Frozen and Dried Medicinal Plants

Three solvents, i.e., water (W), 50% ethanol (*v*/*v*) (E), and dimethyl sulfoxide (D), were tested to select suitable extractants for isolation of the functional components such as phenolic acids, flavonoids, colorants, antioxidants and elements from frozen and dried medicinal plants. Based on the results, we can conclude that the effectiveness of the solvent was dependent on which bioactive compound was isolated. At the same time, the extraction efficiency was significantly influenced by the post-harvest treatment of medicinal plants.

In the case of frozen samples, DMSO seemed to be more efficient for the extraction of phenolic compounds, antioxidants, and colorants. The concentrations of total phenolics (TPC), total flavonoids (TFC), hesperidin, quercetin, color parameters such as yellowness/blueness (b*), chroma (C*), hue angle (h°), and browning index (BI), and total antioxidant activity (TAA) decreased in the order D > E ≥ W. On the other hand, water was a more efficient solvent for the extraction of individual macro- and microelements, as concentrations of Ca, Cu, Fe, K, Mg, Mn, Na, and P decreased in the direction W > E ≥ D (Table 1).

Regarding the dried samples (Table 2), differences between the aprotic (DMSO) and protic systems in terms of extraction efficiency were unambiguous. DMSO appeared to be more suitable for the extraction of colorants than the protic solvents. In general, color parameters b*, C*, h°, and BI decreased in the direction D > E = W. On the contrary, DMSO was less efficient than protic solvents for extracting antioxidants (%RS values), caffeic acid, and macro- and microelements from dried samples (Table 2). Similarities in extraction efficiency among solvents were observed in the concentration of phenolic compounds (TPC, TAA, concentrations of individual phenolic acids, and flavonoids). Regardless of the post-harvest treatment, 50% (*v*/*v*) ethanol was more appropriate for flavonoid extraction compared to water. When comparing W and E, apart from the difference in TFC, the extracts differed even in the concentration of individual macro- and microelements, which were preferably extracted in water for both types of samples.

Drying and extraction of medicinal plants with 50% (*v*/*v*) ethanol was the most appropriate treatment to ensure high yields of the majority of compounds (Supplementary Table S1). The observed differences among solvents were principally related to their different characteristics, mostly polarity, and thus, the solubility of individual compounds in the extraction solvents [19]. Only a limited number of studies dealt with the topic of extraction of bioactive compounds with DMSO. These studies confirmed our findings and indicated DMSO’s suitability for extraction of phenolic compounds [7,20] and colorants [21] from fresh/frozen fruit and herbal materials. However, there are no published data regarding the application of DMSO for dried herbal extraction. Due to DMSO hygroscopicity, higher residual moisture in the frozen samples can increase the permeability of cell tissue and thus enable better mass transfer by molecular diffusion as well as the recovery of water-soluble bioactive compounds. In the case of dried samples, structural and physical changes in the surface of plants could occur, e.g., the formation of a crust, which defended the solvent penetration into plant material. Furthermore, due to lower water content in dried samples, DMSO was not diluted, and some studies showed that pure organic solvents were less efficient than binary solvent mixtures [22,23]. Our study also confirmed the low efficiency of water for the extraction of phenolic compounds and antioxidants and, at the same time, its higher suitability for element extraction. These results are in accordance with previous findings for different plants [19,23,24,25,26].

**Figure 1:**
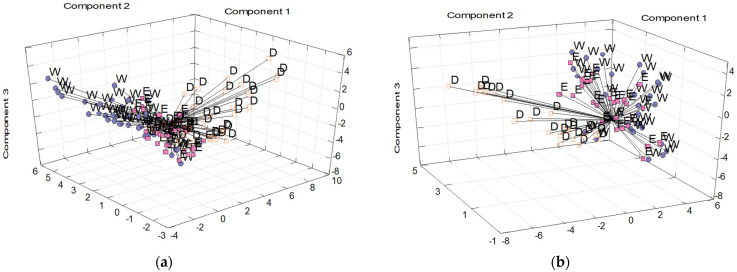
Principal component analysis of (**a**) frozen and (**b**) dried medicinal plants according to extraction solvent used (W—water; E—50% (*v*/*v*) ethanol; D—dimethyl sulfoxide). All original experimental parameters were used for principal components construction.

The obtained dataset of experimental characteristics of medicinal plants, altogether 32 parameters, was used for discrimination and classification of samples according to extraction solvent by means of pattern recognition multivariate statistics. First, principal component analysis (PCA) was applied to visualize the differences/similarities among individual herbal extracts and their clustering tendency and to study the main sources of its variability. The PCA results (Figure 1) proved that plant extracts could be differentiated according to the extraction solvent, as three clusters of vectors (not clearly differentiated) are obvious on the plot of vector scores for both frozen and dried medicinal plants.

In the case of frozen samples (Figure 1a), the differentiation capability seemed to be more distinctive. Applying PCA to the dataset of experimental characteristics of frozen herbs, the first four principal components (PC) explained cumulatively 54.2% of the whole system variability. Parameters TFC, b*, and C* played a dominant role in PC1 construction, and thus, it could be concluded that these parameters describe the maximum of the dataset variability.

In the case of dried samples (Figure 1b), there was a considerable clustering tendency. Samples were clustered into two general subgroups according to the types of solvents: aprotic (D) and protic (E and W). The first four principal components cumulatively explained 56.7% of the system variability. Concentrations of K and P had the most significant weight in PC1, whereas TPC and TFC values were in PC2.

**Figure 2:**
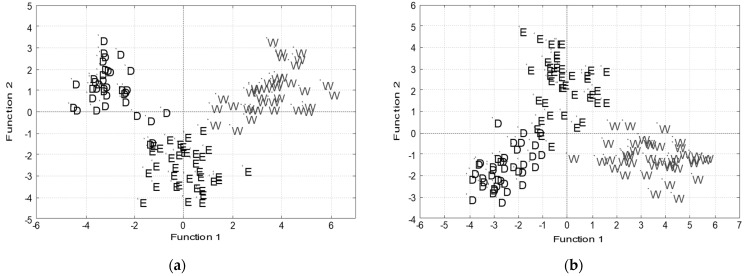
Canonical discriminant analysis of (**a**) frozen and (**b**) dried medicinal plants according to extraction solvent used (W—water; E—50% (*v*/*v*) ethanol; D—dimethyl sulfoxide). Thirty-two original experimental parameters were used for discriminant function construction.

The results of canonical discriminant analysis (CDA) according to the extraction solvents for frozen (Figure 2a) and dried samples (Figure 2b) indicated the discrimination of samples into three discrete zones. The water extracts differed the most from 50% (*v*/*v*) ethanol and DMSO extracts, although—as expected—all three types of extracts were different from each other. These results correlated well with solvent properties, particularly with their polarity. The discrimination of frozen and dried herbal samples according to the extraction solvent possessed 97.2, resp. 97.5% correctness. Color parameters b*, C*, and BI were the most significant parameters for discrimination function construction. The method of kth nearest neighbor analysis distinguished individual extraction systems for both frozen and dried samples with 100% accuracy for k = 1. The qualitative properties of herbal extracts were significantly different.

### 2.2. Effect of Post-Harvest Treatment, Geographical Origin, and Production Year on Characteristics of Frozen and Dried Medicinal Plants

In order to identify the statistically significant descriptors for characterization and differentiation of the water, 50% (*v*/*v*) ethanol, and DMSO herbal extracts, individual experimental characteristics were processed using ANOVA. The following factors: post-harvest processing (freezing versus drying), geographical location (Brno versus Lednice), and production year (2015 versus 2016) were used for mutual comparison.

As is obvious from Table 3, statistically significant differences in many monitored parameters (15 of 32 for water extracts, 16 of 32 for ethanol extracts, and 13 of 32 for DMSO extracts) were found in the case of evaluation of post-harvest treatment effects on properties of herbal extracts. Generally, higher concentrations of the monitored characteristics were determined in dried herbs in comparison with frozen ones for protic systems, while opposite trends were found for aprotic solvent DMSO.

The results for protic systems were in good agreement with the previous study of Kouřimská et al. (2016), who pointed out that probably higher levels of phytochemicals in dried samples (drying at 30 °C) could be caused by a slow decrease of water content during drying which acted as a stress factor for the plant, thereby causing defensive metabolic processes such as the shikimate pathway to form phenolic compounds [15]. Previous studies confirmed that freezing was less appropriate post-harvest treatment when protic solvents such as water, ethanol–water or methanol–water mixtures were used for extraction, suggesting that freezing could cause some damage to phytochemicals soluble in polar protic solvents induced by the formation of ice crystals [1,15,27,28]. The formation of ice crystals probably had the main effect on the decrease of bioactive compounds. The level of damage is dependent on the freezing rate, the final temperature of the frozen plant, choice of species, variety, or size [29,30]. In addition, enzymatic degradation of phenolic compounds during the processing of fresh medicinal plants could be expected, whereas the enzymes were active [27]. Further structural and physical changes in the plant material could be expected due to processing, e.g., coloring/decoloring, crust formation, and inactivation of bacteria and enzymes, which influenced the final extraction process [31].

In the case of DMSO, the extraction process was, as finally expected, influenced by its hygroscopicity, its ability to penetrate the cell membranes as well as the residual moisture of the medicinal plant material. Due to the DMSO membrane penetration tendency, extraction processes could take place more quickly, which probably happened with the frozen matrix. In the case of dried samples extraction by DMSO, it was expected that some phytochemicals soluble in this solvent could be degraded or bio-transformed due to increased temperature, which may cause a decrease of some phytochemicals in dried matrix.

**Figure 3:**
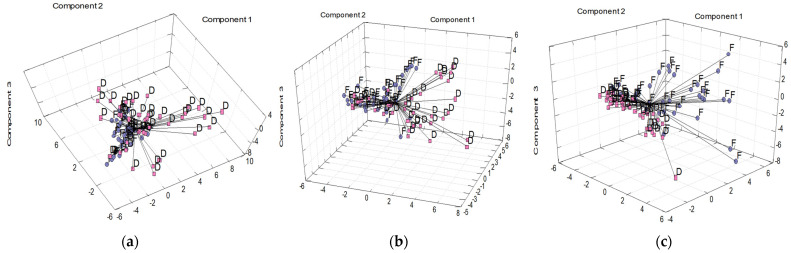
Differentiation of (**a**) water, (**b**) 50% (*v*/*v*) ethanol, and (**c**) dimethyl sulfoxide extracts according to the post-harvest treatment (F—freezing; D—drying) using principal component analysis. Thirty-two original experimental parameters were used for principal components construction.

PCA analysis (Figure 3a–c), performed for each solvent system separately, confirmed the observed trends—significant differences between the frozen and dried herbal samples in functional components composition. The performed evaluation indicated either partial or absolute differentiation of vectors into two clusters according to the post-harvest treatment of herbal samples. The first four components cumulatively explain 59.2, 56.0, and 60.2% of the variability of the whole system for W, E, and D extracts, respectively. Results of PCA indicated that in PC1 constructions, parameters TPC, TAA, and b* for water extracts; TFC, b*, and C* for 50% (*v*/*v*) ethanol extracts; and TPC, TFC, and TAA for DMSO extracts; played a dominant role. Discrimination analysis based on CDA and kth nearest neighbor analysis (Table 4) resulted in high classification scores of 97.4–100% and 81.6–100% for each extraction system. The post-harvest treatment had the dominant effect on extract properties. Despite a slightly lower discrimination accuracy, the results obtained are in accordance with our previous study, where an absolute classification of ethanolic extracts of frozen and dried medicinal plants was obtained [1]. In the case of geographical origin, non-significant differences (*p* < 0.05) were observed for the majority of the evaluated parameters, except for Na and K (Table 3), suggesting the geographical similarities of both localities. Brno and Lednice are 50 km from each other, so similar climatic conditions were expected. Previous studies affirmed that temperature, precipitation amount, altitude, frost-free period, sunshine duration, soil pH, soil organic matter, and available K in the soil could affect bioactive compound concentration [32,33,34]. As follows from meteorological data (refer to Section 3.2), the average temperature and amount of precipitations were comparable in both localities. Slight differences in element content might be related to different qualities of the soil in individual botanical gardens. However, it should be noted here that soil analysis was not the subject of the study. The recognitions of localities were satisfactory, although the classification scores were lower (82.5–85.5%, Table 4) than other criteria. Parameters b*, C*, and BI were identified as the most important for the geographical differentiation of extracts, without respect to the type of solvent. The recognition of localities was comparable to that observed for the discrimination of medicinal plants according to the plant families [1].

**Figure 4:**
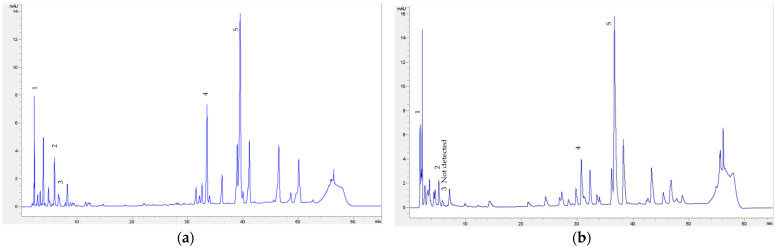
Comparison of chromatograms of phenolic compounds (1—gallic acid, 2—chlorogenic acid, 3—caffeic acid, 4—rutin, and 5—myricetin) identified in ethanolic extracts of frozen Galega officinalis harvested in (**a**) 2015 and (**b**) 2016 at the Medicinal Herbs Centre in Brno.

Similarly, in the case of production years’ comparison (2015 versus 2016), only slight differences were found for most parameters. Certain differences were noted in the element’s concentration and phenolic acids composition (Table 3). Generally, quantitatively higher concentrations of some elements (Cu, Fe, Na, Al), phenolic compounds (caffeic, p-coumaric acid), and antioxidants (%RS values) were found in herbs from the 2015 season. The differences between the two production years are evident in Figure 4, which shows a comparison of chromatograms of phenolic compounds identified in ethanolic extracts of frozen *Galega officinalis* harvested at the Medicinal Herbs Centre in Brno in 2015 and 2016. As shown in Figure 4a, the concentrations of phenolic compounds were higher in 2015, and at the same time, caffeic acid was not detected in 2016 (Figure 4b). From meteorological data followed that the average temperatures were similar in both production years; the differences were mostly identified in total precipitation amount; there was more precipitation in 2016. The annual average precipitation was the most important discrimination factor; high precipitation amounts negatively affected the content of bioactive ingredients. CDA possessed high correct classification of extracts (96.1–100%, Table 4), considering the year of production. Parameters of TPC, TAA, b*, C*, and BI represent the most significant discrimination markers. Seasonal fluctuations in functional components corresponded well with the previous study for nettle leaves [35]. Multivariate statistical methods (PCA, CDA) allowed reducing the number of markers responsible for the differentiation of medicinal plants according to selected factors. Six markers, namely TPC, TFC, TAA, b*, C*, and BI, from a total of thirty-two experimental markers, were considered the most relevant for medicinal plant differentiation.

## 3. Materials and Methods

### 3.1. Chemicals and Reagents

The following chemicals of analytical and gradient grade purity were used: 2-aminoethyl-diphenylborate, 5,5 dimethyl-1-pyrroline-N-oxide (DMPO), 2,2-azino-bis(3-ethyl-benzothiazoline-6-sulphonic acid) salt/cation radical (ABTS/ABTS^•+^), acetonitrile, dimethyl sulfoxide (DMSO), (±)-catechin, chlorogenic acid, ethanol, ferulic acid, Folin–Ciocalteu phenol reagent, gallic acid, hesperidin, myricetin, p-coumaric acid, sinapic acid, quercetin, and rutin hydrate (Sigma-Aldrich, St. Louis, MO, USA); potassium persulfate, potassium dihydrogen phosphate, sodium hydrogen phosphate (Merck, Darmstadt, Germany); caffeic acid and luteolin (Alfa Aesar, Ward Hill, MA, USA); formic acid, sodium carbonate, sodium hydroxide (Lachema, Brno, Czech Republic), standard solutions of elements concentration 1 g/L (Analytika, Prague, Czech Republic) and deionized water purified by a Milli-Q A10 Gradient (Millipore Corp., Burlington, MA, USA).

### 3.2. Herbal Material

Table 5 summarizes ten different species of medicinal plants under study. Samples were collected from two different experimental gardens: the Medicinal Herbs Centre (MHC) Brno, Czech Republic; 49°18′ N lat., 16°57′ E log., and Faculty of Horticulture of Mendel University in Brno (FHM), Lednice, Czech Republic; 48°47′ N lat., 16°48′ E log.

Herbs were harvested during the summer and autumn of 2015 and 2016. The weather conditions at localities were: in MHC year 2015/2016—average temperature: 17.5/17.3 °C; average rainfall: 193.7/250.2 mm; humidity: 35–70/40–82%; in FHM year 2015/2016—average temperature: 17.7/17.2 °C; average rainfall: 193.1/308.3 mm; humidity: 30–70/42–85%. The harvested herbs were processed in two different ways: freezing at −18 °C followed by storing in polyethylene boxes and air-drying on trays at 30 °C followed by storing in paper bags for a maximum of six months.

### 3.3. Preparation of Herbal Extracts

Deionized water (W), 50% ethanol (*v*/*v*) (E) solution, and DMSO (D) were used for the extraction of functional components (phenolic acids, flavonoids, colorants, etc.) following our own procedure [1,36]. The extraction efficiency was expressed on a dry weight basis due to different water content in analyzed samples. Balances with an infrared dryer, 300 IR120 (Denver Instrument, Göttingen, Germany), were used for dry matter content determination. A sample of herbs (1 g) was ground and spread on aluminum foil and dried at a maximum temperature of 103 °C to the constant weight.

### 3.4. Determination of Total Phenolic Compounds Content, Total Flavonoid Content, and Color Characteristics

An ultraviolet, visible near-infrared (UV-VIS-NIR) spectrophotometer Shimadzu UV-3600 (Shimadzu, Kyoto, Japan) was utilized. Total phenolic compounds content (TPC) was determined by Folin–Ciocalteu’s method [36]. Total flavonoid content (TFC) was determined by the method using 2-aminoethyl-diphenylborate [37]. Color parameters L* (lightness), a* (redness/greenness), b* (yellowness/blueness), C* (chroma), h° (hue angle), and browning index (BI) in CIE L*a*b* color system were evaluated from measured absorption spectra, as described previously [1].

### 3.5. Determination of Total Antioxidant Activity

X-band electron paramagnetic resonance (EPR) spectrometer e-scan (Bruker Biospin, Karlsruhe, Germany) was used for the determination of total antioxidant activity (TAA) of herbal extracts. TAA of extracts was monitored by ABTS^•+^ assay (expressed as TAA) [35] and by a method based on termination of the hydroxyl radicals in the presence of DMPO spin trap (expressed as radical scavenging value %RS) [1].

### 3.6. Determination of Individual Phenolic Compounds

High-performance liquid chromatography (HPLC) was used to quantify phenolic acids (gallic, chlorogenic, caffeic, p-coumaric, ferulic, sinapic) and flavonoids (catechin, rutin, quercetin, myricetin, hesperidin, luteolin) of herbal extracts. The phenolic compounds were selected on the basis of previous studies focusing on the identification and quantification of phenolic compounds in selected medicinal plants. Agilent 1260 Infinity instrument (Agilent Technologies, Santa Clara, CA, USA) equipped with a diode array detector (DAD) was used following the procedure developed by our group [1].

### 3.7. Determination of Macro- and Microelements

A group of 10 elements, i.e., Al, Ca, Cu, Fe, K, Mg, Mn, Na, P, and Zn, was analyzed in herbal extracts by inductively coupled plasma optical emission spectrometry (ICP-OES) Horiba Ultima 2 instrument (Horiba Scientific, Paris, France). Herbal extracts were analyzed undiluted for water extracts and diluted with deionized water in a ratio of 1:1 and 1:3 for ethanol and DMSO extracts. Filtration of the sample through a 0.22 µm nylon filter (Agilent Technologies, Santa Clara, CA, USA) was performed before analysis. The instrumental settings are summarized in Table 6.

Adverse changes in the signal during the measurement and matrix effects were corrected using In as an internal standard at a concentration of 0.1 mg/L. The instrument was calibrated by the standard addition method at a calibration range of 0–250 mg/L for Ca, K, Mg, Na, and P and 0–50 mg/L for Al, Cu, Fe, Mn, and Zn, respectively. Recovery of the method was assessed by analysis of spiked samples at two concentration levels (2 and 5 mg/L) using multielement standard solutions. Recoveries obtained for a spiked herbal extract analyzed in the same way as the original samples ranged from 92 to 105%.

### 3.8. Statistical Analysis

The results were expressed as mean value ± standard deviation (n = 4). The statistical analysis was performed using Unistat v. 6.0 software (Unistat Statistical Software Ltd., London, UK). Multiple comparisons were carried out by analysis of variance (ANOVA) Tukey’s HSD procedure at a level of significance *p* ≤ 0.05. The experimental dataset was processed by multivariate statistics involving principal component analysis (PCA), canonical discriminant analysis (CDA), and kth nearest neighbor discriminant analysis to assess the influence of various factors on the monitored parameters of medicinal plants.

## 4. Conclusions

The results obtained revealed that the multi-experimental evaluation of medicinal plants by various methods (spectroscopic and chromatographic) connected with proper chemometric analyses represents an efficient tool to assess the influence of various production and post-production factors on their characteristics. ANOVA and multivariate statistical procedures allowed us to evaluate, visualize, and classify similarities/differences between medicinal plants. High classification scores (61.8–100%) in recognition and prediction ability evaluation indicated that the composition of functional compounds of herbs was significantly influenced by post-harvest processing and seasonal and geographical factors. From all of them, post-harvest processing and choice of extraction solvent influenced the properties of samples most significantly. Six important markers (TPC, TFC, TAA, b*, C*, BI) responsible for the differentiation of medicinal plants according to selected criteria were identified.

## Figures and Tables

**Table 1:** Statistically significant (*p* < 0.05) descriptors between frozen medicinal plants according to solvents selected by ANOVA Tukey’s HSD (analysis of variance—Tukey’s honestly significant difference test), without respect to plant species, locality, and year of production.

Parameter	Comparisons *	Difference	Standard Error	Q Stat	Probability	Parameter
TPC	D–W	25.2	5.0	7.1	0.0000	D > E = W
	D–E	17.5	5.0	4.9	0.0020	
TFC	D–W	28.2	4.6	8.8	0.0000	D > E >W
	E–W	11.1	4.6	3.5	0.0428	
	D–E	17.1	4.6	5.3	0.0009	
L*	E–D	0.9	0.1	8.6	0.0000	E = W > D
	W–D	0.6	0.1	5.5	0.0006	
a*	W–D	1.8	0.2	10.4	0.0000	W = E > D
	E–D	1.6	0.2	8.9	0.0000	
b*	D–E	5.3	0.6	11.7	0.0000	D > E = W
	D–W	0.3	0.6	0.6	0.0000	
C*	D–E	5.4	0.7	11.5	0.0000	D > E = W
	D–W	0.2	0.7	0.5	0.0000	
h°	D–W	17.7	1.9	13.1	0.0000	D > E > W
	E–W	11.3	1.9	8.3	0.0000	
	D–E	6.4	1.9	4.8	0.0000	
BI	D–E	4.5	0.6	10.5	0.0000	D > E = W
	D–W	4.0	0.6	9.3	0.0000	
TAA	D–W	133.5	27.3	6.9	0.0000	D > E = W
	D–E	91.2	27.3	4.7	0.0033	
%RS	W–E	44.3	11.7	5.4	0.0007	W > E = D
Ca	W–D	4302.2	541.2	11.2	0.0000	W > E = D
	W–E	4013.3	541.2	10.5	0.0000	
Cu	W–E	8.4	2.0	6.0	0.0001	W > E = D
	W–D	8.2	2.0	5.8	0.0002	
Fe	W–E	4.3	0.8	8.0	0.0000	W > E = D
	W–D	3.6	0.8	6.7	0.0000	
K	W–D	5250.5	1002.4	7.4	0.0000	W > E = D
	W–E	5008.9	1002.4	7.1	0.0000	
Mg	W–D	1421.7	195.0	10.3	0.0000	W > E > D
	E–D	526.1	195.0	3.8	0.0220	
	W–E	895.5	195.0	6.5	0.0000	
Mn	W–D	7.1	1.0	9.9	0.0000	W > E = D
	W–E	6.1	1.0	8.5	0.0000	
Na	W–D	736.4	163.3	6.4	0.0001	W > E = D
	W–E	690.0	163.3	6.0	0.0002	
P	W–D	1237.4	184.7	9.5	0.0000	W = E > D
	E–D	916.1	184.7	7.0	0.0000	
Zn	E–D	14.8	2.2	9.6	0.0000	D > W > E
	W–E	7.3	2.2	4.7	0.0033	
	D–W	7.5	2.2	4.9	0.0023	
hesperidin	D–W	10,420.0	4159.9	3.5	0.0368	D > E = W
	D–E	10,123.6	4159.9	3.4	0.0436	
quercetin	D–W	257.8	105.2	3.5	0.0419	D ≥ E = W

* Notation X–Y indicates that parameter X > Y. W—deionized water, E—50% (*v*/*v*) ethanol, D—dimethyl sulfoxide, TPC—total polyphenol content, TFC—total flavonoid content, L*—lightness, a*—redness/greenness, b*—yellowness/blueness, C*—chroma, h°—hue angle, BI—browning index, TAA—total antioxidant activity, %RS—the percentage of scavenged radicals.

**Table 2:** Statistically significant (*p* < 0.05) descriptors between dried medicinal plants according to solvents selected by ANOVA Tukey’s HSD (without respect to plant species, locality, and year of production).

Parameter	Comparisons *	Difference	Standard Error	Q Stat	Probability	Parameter
TFC	E–W	15.9	5.1	4.5	0.0058	E ≥ D = W
L*	E–D	0.8	0.1	4.4	0.0066	E > W = D
	E–W	0.7	0.1	4.2	0.0101	
a*	W–D	2.2	0.3	11.6	0.0000	W = E > D
	E–D	1.8	0.3	9.5	0.0000	
b*	D–E	4.5	0.8	8.3	0.0000	D > E = W
	D–W	3.4	0.8	6.1	0.0001	
C*	D–E	4.8	0.8	8.4	0.0000	D > E = W
	D–W	3.7	0.8	6.5	0.0000	
h°	D–W	12.2	2.3	7.5	0.0000	D > E = W
	D–E	8.0	2.3	4.9	0.0022	
BI	D–E	3.6	0.7	7.1	0.0000	D > E = W
	D–W	2.0	0.7	4.0	0.0145	
%RS	W–D	46.0	8.7	7.5	0.0000	W = E > D
	E–D	45.0	8.7	7.3	0.0000	
Al	E–D	5.8	1.0	8.5	0.0000	W = E > D
	W–D	5.6	1.0	8.2	0.0000	
Ca	W–D	5933.5	666.8	12.6	0.0000	W > E = D
	W–E	4712.3	666.8	10.0	0.0000	
Cu	E–D	2.7	0.7	5.5	0.0004	E = W ≥ D
Fe	W–D	3.3	0.7	6.9	0.0000	W > E = D
	W–E	1.9	0.7	3.9	0.0191	
K	E–D	11,877.3	1278.0	13.1	0.0000	E = W > D
	W–D	11,540.7	1278.0	12.8	0.0000	
Mg	W–D	1996.3	210.0	13.4	0.0000	W = E > D
	E–D	1532.0	210.0	10.3	0.0000	
Mn	W–D	14.3	2.0	10.1	0.0000	W > E ≥ D
	W–E	10.3	2.0	7.3	0.0000	
Na	W–D	546.3	147.0	5.3	0.0009	W > E ≥ D
	E–D	390.3	147.0	3.8	0.0243	
P	W–D	2028.9	169.0	17.0	0.0000	W > E > D
	E–D	857.6	169.0	7.2	0.0000	
	W–E	1171.3	169.0	9.8	0.0000	
Zn	W–D	7.7	1.9	5.8	0.0002	W = E > D
	E–D	2.8	1.9	3.7	0.0275	
caffeic acid	W–D	185.8	45.4	5.8	0.0002	W = E > D
	E–D	110.3	45.4	3.4	0.0436	

* Notation X–Y indicates that parameter X > Y. W—deionized water, E—50% (*v*/*v*) ethanol, D—dimethyl sulfoxide, TPC—total polyphenol content, TFC—total flavonoid content, L*—lightness, a*—redness/greenness, b*—yellowness/blueness, C*—chroma, h°—hue angle, BI—browning index, TAA—total antioxidant activity, %RS—percentage of scavenged radicals.

**Table 3:** Statistically significant (*p* < 0.05) differences in monitored parameters of water, 50% (*v*/*v*) ethanol and DMSO extracts from frozen and dried medicinal plants produced during two years (2015 and 2016) and originated from two different geographical localities (Brno and Lednice) performed by ANOVA Tukey’s HSD statistical evaluation.

Parameter	Water Extracts				50% (*v*/*v*) Ethanol Extracts				DMSO Extracts			
	Comparison *	Difference	Standard Error	Probability	Comparison *	Difference	Standard Error	Probability	Comparison *	Difference	Standard Error	Probability
*Post-harvest treatment*												
TPC	D–F	12.8	4.3	0.0038	D–F	11.6	5.4	0.0333	F–D	16.2	6.4	0.0139
TFC	D–F	8.2	2.8	0.0038	D–F	13.0	5.1	0.0132	-	-	-	-
L*	F–D	0.9	0.2	0.0002	F–D	0.5	0.1	0.0001	-	-	-	-
a*	-	-	-	-	F–D	0.2	0.1	0.0252	-	-	-	-
b*	D–F	2.0	0.4	0.0001	D–F	1.1	0.3	0.0003	-	-	-	-
C*	D–F	2.0	0.4	0.0000	D–F	1.1	0.3	0.0003	-	-	-	-
h°	D–F	4.2	1.5	0.0064	-	-	-	-	-	-	-	-
BI	D–F	2.0	0.5	0.0001	D–F	1.0	0.3	0.0008	-	-	-	-
TAA	D–F	89.5	23.3	0.0003	D–F	87.7	31.7	0.0072	-	-	-	-
%RS	-	-	-	-	-	-	-	-	-	-	-	-
Al	-	-	-	-	-	-	-	-	F–D	3.2	0.6	0.0000
Fe	D–F	1.8	0.6	0.0410	-	-	-	-	F–D	1.5	0.6	0.0154
Mn	D–F	7.0	2.7	0.0108	D–F	671.6	203.7	0.0015	-	-	-	-
Na	D–F	452.8	222.2	0.0452	-	-	-	-	F–D	262.7	70.9	0.0004
P	D–F	536.8	207.6	0.0117	-	-	-	-	F–D	254.7	30.0	0.0000
Cu	-	-	-	-	D–F	1.9	0.6	0.0013	F–D	9.2	2.3	0.0001
K	-	-	-	-	D–F	6001.7	1337.5	0.0000	F–D	5634.0	765.0	0.0000
Mg	-	-	-	-	D–F	671.6	203.7	0.0015	F–D	334.3	71.0	0.0000
Zn	-	-	-	-	D–F	6.0	2.3	0.0093	F–D	13.7	1.5	0.0000
gallic acid	-	-	-	-	-	-	-	-	F–D	61.2	30.0	0.0450
chlorogenic acid	D–F	146.1	60.8	0.0188	D–F	175.5	71.1	0.0160	-	-	-	-
caffeic acid	D–F	166.4	53.8	0.0028	D–F	75.7	32.6	0.0228	F–D	61.2	30.0	0.0020
ferulic acid	-	-	-	-	-	-	-	-	F–D	159.2	67.3	0.0020
quercetin	D–F	168.3	70.5	0.0196	-	-	-	-	F–D	77.0	24.0	0.0207
hesperidin	-	-	-	-	D–F	4260.5	1885.5	0.0268	-	-	-	-
*Geographical origin*												
K	L–B	3065.1	1257.1	0.0172	L–B	3363.7	1454.8	0.0236	L–B	3259.5	1455.8	0.0305
Na	L–B	646.5	215.3	0.0037	L–B	351.9	126.3	0.0068	L–B	156.7	74.9	0.0399
*Year of production*												
%RS	-	-	-	-	15–16	73.3	11.5	0.0000	15–16	43.8	8.2	0.0000
Al	-	-	-	-	15–16	14.3	1.2	0.0000	15–16	1.8	0.7	0:0085
Cu	15–16	1.7	0.8	0.0391	15–16	1.2	0.6	0.0406	15–16	12.6	2.0	0.0000
Fe	15–16	2.3	0.8	0.0087	15–16	3.5	0.5	0.0000	-	-	-	
Na	15–16	531.0	219.6	0.0180	15–16	519.5	216.7	0.0191	15–16	224.6	72.5	0.0028
caffeic acid	-	-	-	-	15–16	73.7	32.6	0.0267	15–16	52.2	24.9	0.0392
*p*-coumaric acid	-	-	-	-	15–16	1.7	0.8	0.0445	-	-	-	

* Notation X–Y indicates that parameter X > Y. D—dried medicinal plants, F—frozen medicinal plants, B—Brno, L—Lednice, 15—year 2015, 16—year 2016, TPC—total polyphenol content, TFC—total flavonoid content, L*—lightness, a*—redness/greenness, b*—yellowness/blueness, C*—chroma, h°—hue angle, BI—browning index, TAA—total antioxidant activity, %RS—percentage of scavenged radicals.

**Table 4:** Classification scores of water, 50% (*v*/*v*) ethanol, and DMSO herbal extracts using methods of canonical discriminant analysis (CDA) and kth nearest neighbor discriminant analysis. Extracts were classified using different classification criteria—extraction solvent, post-harvest processing, geographical origin of plant production, as well as production year.

Discriminant Method		Solvent	Processing	Origin	Year
CDA		Water	97.4%	84.2%	96.1%
		50% (*v*/*v*) ethanol	98.7%	85.5%	100%
		Dimethyl sulfoxide	100%	82.9%	96.1%
kth neighbor	k = 1	Water	100%	100%	100%
		50% (*v*/*v*) ethanol	100%	100%	100%
		Dimethyl sulfoxide	100%	100%	100%
	k = 2	Water	81.6%	65.8%	80.3%
		50% (*v*/*v*) ethanol	89.5%	75.0%	84.2%
		Dimethyl sulfoxide	96.1%	61.8%	86.8%

**Table 5:** Investigated medicinal plant species.

Botanical Name	Part Used	Processing	Locality	Production Year
*Lavandula angustifolia*	Flower	Freezing/drying	Brno/Lednice	2015, 2016
*Salvia sclarea*	Flower	Freezing/drying	Brno/Lednice	2015, 2016
*Salvia officinalis*	Leaf	Freezing/drying	Brno/Lednice	2015, 2016
*Melissa officinalis*	Leaf	Freezing/drying	Brno/Lednice	2015, 2016
*Hyssopus officinalis*	Flower	Freezing/drying	Brno/Lednice	2015, 2016
*Mentha piperita*	Leaf	Freezing/drying	Brno/Lednice	2015, 2016
*Hypericum perforatum*	Flower	Freezing/drying	Brno/Lednice	2015, 2016
*Galega officinalis*	Flower	Freezing/drying	Brno/Lednice	2015, 2016
*Calendula officinalis*	Flower	Freezing/drying	Brno/Lednice	2015, 2016
*Silybum marianum*	Seed	Drying	Brno/Lednice	2015, 2016

**Table 6:** The operating condition of the Agilent ICP-OES for analysis of elements in plant extracts.

Element	Wavelength [nm]	Input Slot [µm]	Output Slot [µm]
Al	396.152	20	15
Ca	422.673	20	15
Cu	327.396	20	15
Fe	259.940	20	15
K	766.490	20	15
Mg	285.213	20	15
Mn	257.610	20	15
Na	588.995	20	15
P	213.618	20	15
Zn	206.191	20	15
** Parameter **	** Water **	** 50% (*v*/*v*) ethanol **	** DMSO **
RF power	1350 W	1400 W	1350 W
Plasma gas	13 L/min	12.5 L/min	12.5 L/min
Auxiliary gas	0.1 L/min	0.1 L/min	0.1 L/min
Nebulizer gas	0.85 L/min	0.85 L/min	0.85 L/min
Heath gas	0.2 L/min	0.5 L/min	0.5 L/min
Nebulizer pressure	3 bar	3 bar	3 bar

## Data Availability

The data presented in this study are available in Supplementary Table S1.

## Supplementary Materials

The following supporting information can be downloaded at: https://www.mdpi.com/article/10.3390/molecules28104075/s1, Table S1: Analytical results (mean ± SD) of aqueous, 50% (*v*/*v*) ethanolic and DMSO extracts prepared from frozen and dried medicinal plants originating from Brno and Lednice produced during two years (2015 and 2016).
